# Dynamic Equilibrium of the Aurora A Kinase Activation Loop Revealed by Single‐Molecule Spectroscopy

**DOI:** 10.1002/anie.201704654

**Published:** 2017-08-07

**Authors:** James A. H. Gilburt, Hajrah Sarkar, Peter Sheldrake, Julian Blagg, Liming Ying, Charlotte A. Dodson

**Affiliations:** ^1^ National Heart & Lung Institute SAF Building Imperial College London London SW7 2AZ UK; ^2^ Cancer Research UK Cancer Therapeutics Unit The Institute of Cancer Research 15 Cotswold Road Sutton Surrey SM2 5NG UK

**Keywords:** activation loop, Aurora A, drug design, kinases, single-molecule studies

## Abstract

The conformation of the activation loop (T‐loop) of protein kinases underlies enzymatic activity and influences the binding of small‐molecule inhibitors. By using single‐molecule fluorescence spectroscopy, we have determined that phosphorylated Aurora A kinase is in dynamic equilibrium between a DFG‐in‐like active T‐loop conformation and a DFG‐out‐like inactive conformation, and have measured the rate constants of interconversion. Addition of the Aurora A activating protein TPX2 shifts the equilibrium towards an active T‐loop conformation whereas addition of the inhibitors MLN8054 and CD532 favors an inactive T‐loop. We show that Aurora A binds TPX2 and MLN8054 simultaneously and provide a new model for kinase conformational behavior. Our approach will enable conformation‐specific effects to be integrated into inhibitor discovery across the kinome, and we outline some immediate consequences for structure‐based drug discovery.

Protein kinases are essential for the regulation and signaling of eukaryotic cells and are important drug targets in cancer and inflammatory disease.^1^ Many kinases are regulated by phosphorylation of a regulatory Ser/Thr/Tyr residue on a region of the kinase known as the activation loop or T‐loop. The influence of phosphorylation and interactions with small‐molecule inhibitors on kinase conformation can be summarized by two models. In the first model, phosphorylation achieves activation by “locking” the activation loop in a conformation where the catalytic residues are aligned (Figure 1 a).^2^ In the second model, an inactive‐conformation kinase bound to a type II inhibitor (an inhibitor whose binding site extends into a specific allosteric pocket adjacent to the ATP‐binding site) is in equilibrium with the ligand‐free kinase in an active conformation (Figure 1 b). In the context of these models, the active conformation is typified by the activation loop being oriented to form the protein substrate binding site and the aspartic acid of the conserved DFG motif at the beginning of this loop pointing into the ATP binding site to coordinate Mg^2+^/ATP (DFG‐in). In the classical inactive conformation, both the activation loop and the DFG motif are rotated by 180°, and a phenylalanine replaces the aspartic acid in the ATP‐binding pocket (DFG‐out). Both models have been supported by X‐ray crystallography but direct experimental testing of kinase activation loop mobility has proved impossible to date as activation loop dynamics occur on the same timescale as NMR intermediate exchange.^3^

**Figure 1:**
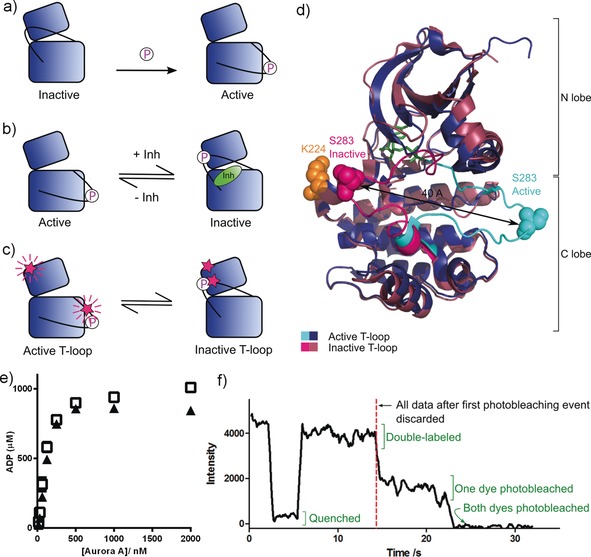
a) Current model of kinase activation: Phosphorylation on the activation loop (black line) locks the kinase in an active T‐loop conformation. b) Current equilibrium model of type II inhibitor binding: Active apo kinase is in equilibrium with inhibited kinase in an inactive T‐loop conformation. c) Proposed equilibrium model showing TMR‐labeled sites (pink stars) and the expected fluorescence signal. d) Conformational change and labeling sites in Aurora A. Active T‐loop: blue, inactive T‐loop: pink. Activation loop shown in cyan/bright pink. Labeling sites (K224 in orange and S283 in pink/cyan) shown as filled spheres. PDBs 1OL5 and 2WTV. e) Kinase activity assay for unlabeled pseudo‐wildtype Aurora A (□) and TMR‐labeled K224C/S283C (▴). Activity shown as the [ADP] produced over the course of a 1 h reaction. The difference in activity between pseudo‐wildtype and labeled protein cannot be accounted for by incomplete protein labeling (see the Supporting Information for labeling efficiency). f) Example trace of a double‐labeled K224C/S283C Aurora A single molecule showing the background‐subtracted fluorescence intensity over time.

To test these models directly, we used single‐molecule fluorescence spectroscopy to monitor the conformation of the kinase activation loop. We implemented our assay in Aurora A kinase, a mitotic kinase whose catalytic activity can be increased in the presence of its protein binding partner TPX2^4^ and inhibited with numerous drug‐like small molecules.4d, 5 Interest in Aurora A and its conformational plasticity has recently increased owing to the discovery that inhibiting Aurora A with MLN8054 or CD532, both observed to bind Aurora A in an inactive T‐loop conformation, disrupts the interaction of Aurora A with the proto‐oncogenic transcription factor *N*‐Myc. This leads to degradation of *N*‐Myc and offers an alternative therapeutic strategy for *N*‐Myc‐driven tumors (currently under clinical evaluation with MLN8237/alisertib).5d, 6 These factors make Aurora A an excellent clinically relevant system for studying the heterogeneity and dynamics of T‐loop conformation underlying the observed structural and catalytic properties of protein kinases.

For clarity, we use the terms “active T‐loop” and “inactive T‐loop” to refer to the two orientations of the activation loop (Figure 1 c, d; see also the Supporting Information, Figure S1a). We reserve the terms DFG‐in and DFG‐out for discussions of the detailed orientation of the DFG motif (Figure S1 b).

We labeled a pseudo‐wildtype construct of Aurora A (C290A/C393A)^7^ with tetramethylrhodamine (TMR) on cysteine residues introduced into the activation loop (S283C) and the N lobe of the kinase (K224C; Figure 1 d). The fluorescence of TMR is quenched when two molecules are closer than about 15 Å, and this phenomenon has previously been used to probe small distance changes at the single‐molecule level.^8^ Control reactions confirmed that only two sites were available for coupling (Figure S2).

The catalytic activity of TMR‐labeled phosphorylated Aurora A is similar to that of the unlabeled pseudo‐wildtype protein (Figure 1 e). We tethered labeled protein to a cover slip via a His tag (Figure S4) and imaged the fluorescence intensity by total internal reflection fluorescence (TIRF) microscopy. Single‐molecule fluorescence traces from double‐labeled Aurora A molecules were identified by the presence of two‐step photobleaching at their end.^9^ Before photobleaching, these molecules exhibited a single high fluorescence intensity and transiently entered a low‐intensity, quenched state (example trace in Figure 1 f), which we interpret to indicate changes in the position of the Aurora A T‐loop: high fluorescence indicating an active‐conformation T‐loop, and quenched fluorescence an inactive T‐loop (see the Experimental Methods Section in the Supporting Information).

The fluorescence intensity histogram for phosphorylated Aurora A shows two peaks, indicating that the kinase adopts both active and inactive T‐loop conformations (Figure 2 a). The relative areas of the peaks indicate that in solution the majority of molecules adopt an active T‐loop conformation and a minority an inactive conformation (Table 1). This is consistent with the active T‐loop conformation observed in X‐ray structures of uninhibited kinase and with the high catalytic activity of phosphorylated Aurora A.4b Contrary to the model of a locked phosphorylated T‐loop, our data indicate that the activation loop of phosphorylated Aurora A exists in a dynamic structural equilibrium (*K*_eq_=0.3±0.1).

**Figure 2:**
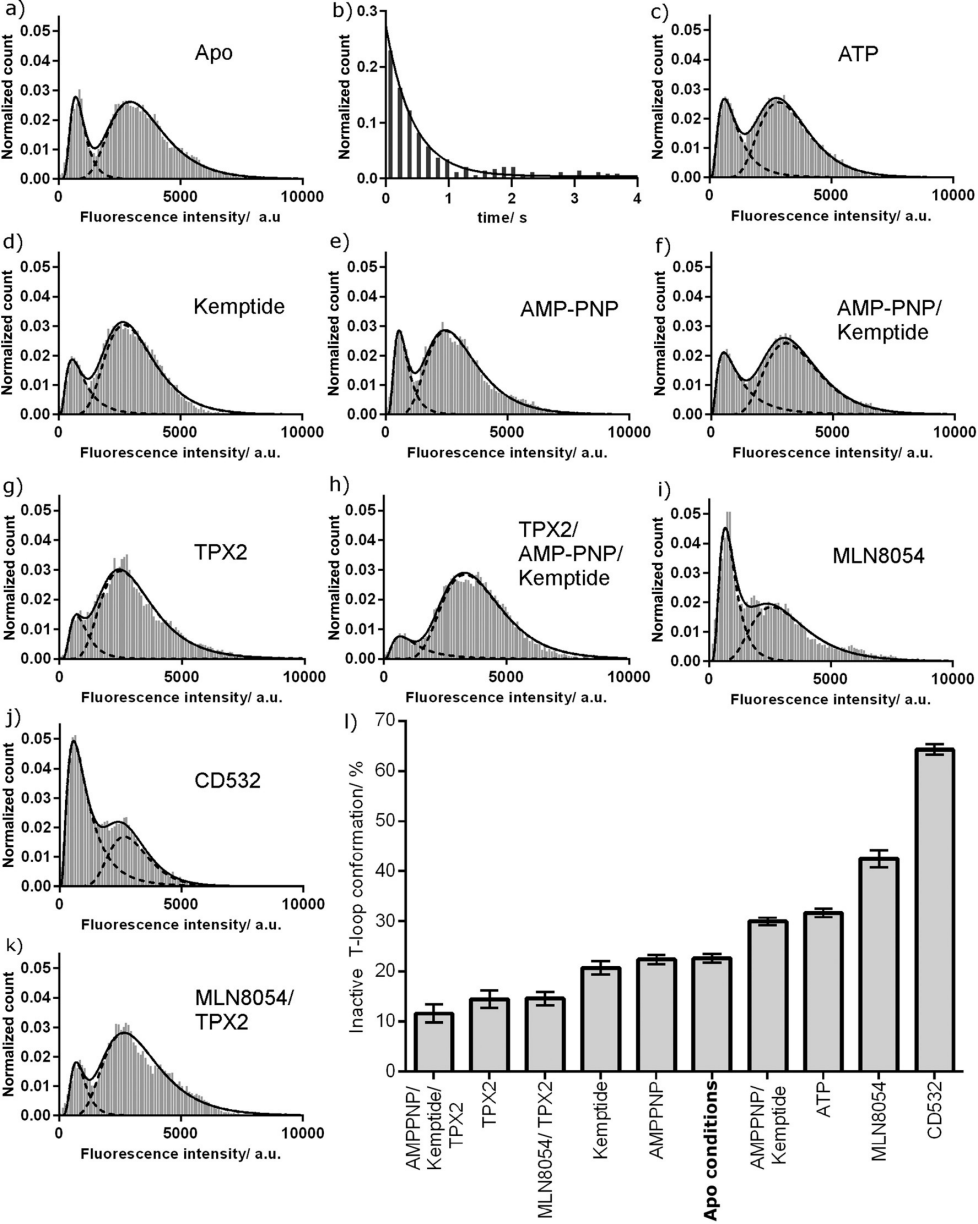
Fluorescence intensity distribution for phosphorylated TMR‐labeled K224C/S283C Aurora A. a) Intensity histogram of unliganded Aurora A. b) Dwell time histogram of the quenched inactive T‐loop conformation. c–k) Fluorescence intensity distributions with c) 1 mm ATP, d) 3 mm kemptide, e) 1 mm AMP‐PNP, f) 1 mm AMP‐PNP and 3 mm kemptide, g) 5 μm TPX2, h) 5 μm TPX2, 1 mm AMPPNP, and 3 mm Kemptide, i) 10 μm MLN8054, j) 10 μm CD532, and k) 5 μm TPX2 and 10 μm MLN8054. l) Summary of the conformational preferences of Aurora A under different conditions. Error bars show propagated fitting errors.

**Table 1:** Occupancy of Aurora A conformations under different conditions.

Ligand(s)	Ligand concentration [μm]	*K*_m_, *K*_i_, or IC_50_ [μm]	Inactive T‐loop [%]^[a]^	Active T‐loop [%]^[a]^	*K*_eq_^[b]^	Δ*G*_inactive–active_ at 25 °C [kcal mol^−1^]^[c]^
Apo	–	–	23	77	0.3	0.7
MLN8054	10	0.0003^[d]^	43	57	0.7	0.2
CD532	10	0.048^[e]^	64	36	1.8	−0.4
TPX2	5	0.01^[f]^	14	86	0.2	1.1
TPX2/MLN8054	5/10	0.01^[f]^/0.0003^[d]^	15	85	0.2	1.0
AMPPNP	1000	ND	22	78	0.3	0.7
ATP	1000	36^[g]^	32	68	0.5	0.5
Kemptide	3000	330^[g]^	21	79	0.3	0.8
AMPPNP/Kemptide	1000/3000	ND/330^[h]^	30	70	0.4	0.5
TPX2/AMPPNP/Kemptide	5/1000/3000	0.01^[f]^/ND/330^[h]^	12	88	0.1	1.2

[a] The error of the loop occupancy is propagated from the fitting error of the histograms and is ≤2. [b] *K*_eq_=[inactive T‐loop]/[active T‐loop]. The error on *K*_eq_ is propagated from the fitting error of the histograms and is <0.1. [c] Δ*G*_inactive–active_=−*RT* ln(*K*_eq_). The error on Δ*G*_inactive–active_ is propagated from fitting errors of the histograms and is ≤0.1 kcal mol^−1^. [d] *K*_i_ from Ref. 5b. [e] IC_50_ from Ref. 5d. [f] EC_50_ from Ref. 4b. [g] *K*_m_ from Ref. 4b. [h] *K*_m_ measured in this study (Figure S3) with an error of ±70 μm. ND=not determined.

It has been suggested that the free energy penalty of interconverting between active and inactive conformations of the tyrosine kinases Src and Abl underlies the selectivity of type II inhibitors such as Imatinib, although this has been challenged.^10^ Consequently, there have been efforts to calculate the free energy difference between these conformations. None of these studies explicitly distinguished between the orientation of the DFG motif and the overall orientation of the T‐loop (usually assumed to be coupled); however, the experimental protocols suggest that they report on the interconversion between DFG‐in active T‐loop and DFG‐out inactive T‐loop conformations.^11^ Our experimental measurements report directly on the interconversion between active and inactive T‐loop conformations (without measuring the orientation of the DFG motif), and the free energy difference that we obtained for phosphorylated Aurora A is 0.7±0.1 kcal mol^−1^ (Δ*G*_inactive–active_). This is very similar to the value of 0.8 kcal mol^−1^ that has been measured for the two unassigned conformations of phosphorylated Erk2^12^ and considerably less than the calculated values for Src.11a,11c

We also measured the microscopic rate constant for adopting an active T‐loop conformation, *k*_active_ (Figure 2 b; *k*_active_=2.3±0.2 s^−1^). We were unable to measure *k*_inactive_ directly as the observation time of individual molecules was limited by photobleaching, but we calculated this to be 0.7±0.2 s^−1^ from *K*_eq_.

To determine how kinase substrates influence the conformation of the activation loop, we measured fluorescence intensity distributions of Aurora A in the presence of saturating levels of ATP, kemptide (a 7‐residue peptide substrate), and AMP‐PNP (a non‐hydrolysable analogue of ATP; Figure 2 c–f). Neither kemptide nor AMP‐PNP changed the position of the equilibrium from that of unliganded kinase while, surprisingly, ATP alone and AMP‐PNP/kemptide both slightly increased the population of the inactive T‐loop conformation (Table 1).

We next measured the intensity distribution of Aurora A in the presence of the activator TPX2 (Figure 2 g). Occupancy of the active T‐loop conformation was increased (Table 1), consistent with the increased catalytic activity of the enzyme. To build a picture of the enzyme poised for maximal activity, we measured the distribution of the Aurora A–TPX2 complex bound to AMP‐PNP and kemptide (Figure 2 h). This adopted a predominantly active T‐loop conformation, similar to that of the Aurora A–TPX2 complex alone.

MLN8054 and CD532 are both nanomolar inhibitors of Aurora A, and X‐ray structures show that each binds in an inactive T‐loop conformation (Figure S1 c, d).5b,5d,5g Although both are referred to as type II inhibitors, neither extends into the allosteric hydrophobic pocket, and neither has been captured binding the kinase with a canonical DFG‐out motif. Aurora A bound to MLN8054 adopts an unusual DFG conformation previously termed DFG‐up,5b and Aurora A bound to CD532 is DFG‐in.5d

To determine the effect of these inhibitors on the activation loop of Aurora A in solution, we repeated our assay in their presence (Figure 2 i, j). Each inhibitor resulted in a large increase in the population of the inactive T‐loop conformation, which is consistent with the crystal structures (Table 1). Control measurements showed that neither inhibitor affected the peak fluorescence intensity of TMR, although peak broadening was observed, particularly for CD532 (Figure S5 and Table S1). The number of fluorescent molecules in the field of view remained constant within each control (35±6 kinase alone, 34±5 with MLN8054; *n*=12).

As TPX2 and MLN8054 move the position of the conformational equilibrium in opposing directions, we wondered how they interacted when present simultaneously. This is a physiologically relevant scenario at the mitotic spindle, and kinetic studies have shown that the presence of TPX2 increases the *K*_i_ value of MLN8054 by a factor of greater than four.5b Similar changes have been observed for VX680 and GSK623906A.4d In the presence of both MLN8054 and TPX2, Aurora A adopted a predominantly active T‐loop conformation, similar to that for TPX2 alone (Figure 2 k and Table 1).

To determine whether this result represents a mixture of binary Aurora A–MLN8054 and Aurora A–TPX2 complexes or population of an Aurora A–TPX2–MLN8054 triple complex, we calculated the expected experimental result for the mixture of binary complexes based on the published affinities of the two ligands4b, 5b (see the Supporting Information). A mixture of binary complexes would result in an inactive T‐loop population of 43 %, which is inconsistent with the experimental result (15 %; Table 1). Our experimental samples must thus contain a triple complex of Aurora A–TPX2–MLN8054. We were unable to quantify the extent of triple complex formation, but to account for the experimental results, it must be the major species and must occupy a mainly active T‐loop conformation.

Our measurements indicate that phosphorylated Aurora A is not locked into a single conformation, and that it spontaneously interconverts between active and inactive T‐loop conformations in solution, even in the presence of saturating quantities of the small‐molecule inhibitors MLN8054 and CD532 or of TPX2. To explain our results, we hypothesized that either the ligand residence time is short [and rearrangement of the activation loop occurred in the unliganded kinase; Equations (1), (2), (3)] or that Aurora A bound to saturating quantities of ligand can interconvert between T‐loop conformations.

(1) $$\mathrm{Active}\vec{\leftarrow }\mathrm{Inactive}\overset{+\mathrm{inh}}{\underset{}{↔}}\mathrm{Inactive}\text{-}\mathrm{inh}$$

(2) $$\mathrm{Active}\vec{\leftarrow }\mathrm{Inactive}\overset{+\mathrm{inh}}{\underset{}{↔}}\mathrm{Inactive}\text{-}\mathrm{inh}\vec{\leftarrow }\mathrm{Inactive}{}^{*}\text{-}\mathrm{inh}$$

(3) $$\mathrm{Active}\vec{\leftarrow }\mathrm{Inactive}\vec{\leftarrow }\mathrm{Inactive}{}^{*}\overset{+\mathrm{inh}}{\underset{}{↔}}\mathrm{Inactive}{}^{*}\text{-}\mathrm{inh}$$

We tested this by using the *K*_eq_ value of the kinase alone and the known ligand *K*_d_ to predict the observed *K*_eq_ value in the presence of ligand (see the Supporting Information). Equation (1), an equivalent scheme for TPX2, and Equations (2) and (3) (which postulate the presence of a second sub‐conformation, inactive*, within the inactive T‐loop conformation) all predicted *K*_eq_ values that were 2–4 orders of magnitude different from those measured. We therefore concluded that ligand‐bound Aurora A interconverts between T‐loop conformations and modelled our data by using Equation 4.
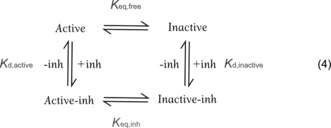



This surprising conclusion is supported by X‐ray crystallography. Three PDB structures (2WTV, 3H10, and 2X81) show Aurora bound to MLN8054 or a similar compound in an inactive T‐loop conformation, while in a fourth, low‐resolution structure (2WTW), the crystal packing is incompatible with an inactive T‐loop, and the MLN8054‐bound kinase adopts an active T‐loop conformation.5b All four Aurora–MLN8054 structures superpose within the N and C lobes of the kinase, varying only in the orientation of the activation loop and in the exact angle between the two lobes. The position of MLN8054 relative to the N lobe is identical across all structures, implying that no change in the binding mode of the inhibitor is required for interconversion between active and inactive T‐loop conformations. We propose that 2WTW and 2WTV represent two snapshots of the Aurora A–MLN8054 conformational ensemble. Interconversion between these two conformations is brought about by movement of the activation loop without the inhibitor leaving the binding site.

To determine whether the reported change in *K*_i_ of MLN8054 upon addition of TPX25b reflects a true change in the *K*_d_ value of MLN8054, or whether it can be accounted for by changes in the position of the conformational equilibrium alone, we partitioned the *K*_i_ value for MLN8054 into conformation‐specific dissociation constants (Equation (4) and the Supporting Information for modeling). In the absence of TPX2, *K*_d,active_=1.0 nm and *K*_d,inactive_=0.4 nm, while in the presence of TPX2, these values are equal and remain unchanged (within experimental accuracy) at 3.3 nm, indicating a genuine change in *K*_d_ for the Aurora A–TPX2 complex.

Our analysis (see the Supporting Information, Equation (S18)) also provides insight into the drivers of inactive and active T‐loop conformations. The position of the equilibrium for any binding partner depends on the *K*_d,active_/*K*_d,inactive_ ratio and the intrinsic position of the equilibrium for the kinase (*K*_eq,free_, which is a constant for each protein). In other words, the proportion of ligand‐bound kinase molecules in an inactive T‐loop conformation is driven by the degree to which an inhibitor can discriminate between inactive and active T‐loop conformations, not by the overall inhibitor–kinase affinity.

Our reported measurements derive from in vitro experiments. While the exact values that we have determined may change in the cellular environment, we expect the principle of conformational equilibria and the models of inhibitor binding that we have established to translate into cell‐based contexts. Our results thus have a number of consequences for drug discovery:

1. At least two Aurora A inhibitors bind a conformation of the kinase (the active T‐loop conformation) that had previously not been expected. It is now possible to measure (and thus develop validated prediction algorithms for) the effect of an inhibitor on kinase conformational equilibria in solution.
2. Potent conformation‐independent inhibitors need to bind both active and inactive T‐loop conformations, and structure‐based drug design may need to focus on common features of these.
3. For inhibitors binding to the inactive T‐loop conformation, differential binding to this conformation should be maximized. This is particularly important for inhibitors designed to induce a specific conformation of the kinase in order to disrupt a physiological interaction (e.g., Aurora A with *N*‐Myc). While the affinity for the active T‐loop conformation is retained, we expect to find a small proportion of the complex (e.g., Aurora A–CD532–*N*‐Myc) in the active T‐loop conformation, even at saturating concentrations of inhibitor.
4. For those kinases where *K*_eq,free_≪1, an inactive T‐loop inhibitor must achieve greater discrimination between active and inactive T‐loop conformations than an inhibitor for a kinase where *K*_eq,free_≥1. Some inhibitors may thus appear to be inactive T‐loop inhibitors when bound to one target and active T‐loop inhibitors when bound to another, potentially contributing to unexpected cellular phenotypes. We predict that the kinase phosphorylation state will also affect the value of *K*_eq,free_.
5. Modification of the target protein (e.g., by binding to a physiological protein partner such as TPX2) can change the binding affinity of an inhibitor beyond what would be predicted solely from a change in the position of the conformational equilibrium. Distinguishing between allosteric partners such as TPX2 and scaffolding partners may be possible from X‐ray structures, from enzyme activity assays, or potentially by inference from physiological function (e.g., catalytic activation vs. substrate recruitment). This means that early decisions over the best form of the enzyme to target are still important.
6. Success in allosteric disruption by using an ATP‐competitive small molecule to displace a conformation‐specific physiological ligand will depend on the properties of the protein–protein interaction to be disrupted (Figure S6). By carefully matching inhibitors and interactions, it may be possible to achieve specificity between different binding partners of the same kinase. In practice, the limits of this approach will need to be found experimentally.

## Conflict of interest

P.S. is a former and J.B. a current employee of The Institute of Cancer Research, which has a commercial interest in the development of Aurora A inhibitors. J.A.H.G., H.S., C.A.D., and L.Y. have no competing financial interests.

## Supporting information

As a service to our authors and readers, this journal provides supporting information supplied by the authors. Such materials are peer reviewed and may be re‐organized for online delivery, but are not copy‐edited or typeset. Technical support issues arising from supporting information (other than missing files) should be addressed to the authors.

Supplementary
