# Supplementary material for: Dynamic Equilibrium of the Aurora A Kinase Activation Loop Revealed by Single‐Molecule Spectroscopy
Source: Angew Chem Int Ed Engl. 2017 Aug 7;56(38):11409–14. doi: 10.1002/anie.201704654 (PMC5601181; doi:10.1002/anie.201704654)
Supplement: Supplementary file 1 — Supplementary [file ANIE-56-11409-s001.pdf]

Angewandte Chemie
Eine Zeitschrift der Gesellschaft Deutscher Chemiker

Supporting Information

Dynamic Equilibrium of the Aurora A Kinase Activation Loop Revealed by Single-Molecule Spectroscopy

*James A. H. Gilburt, Hajrah Sarkar, Peter Sheldrake, Julian Blagg, Liming Ying, and Charlotte A. Dodson**

anie_201704654_sm_miscellaneous_information.pdf

Author Contributions

J.A.H.G., H.S., L.Y., and C.A.D. designed experiments; J.A.H.G. and H.S. carried out experiments; P.S. synthesized CD532; L.Y., J.B. and C.A.D. supervised experimental work; J.A.H.G., H.S. and C.A.D. analyzed data; J.A.H.G. and C.A.D. wrote the paper. All authors commented on the final text.

Supplementary materials and methods:

Reaction scheme modelling **2**

Calculation of the expected population of inactive T-loop conformation in the presence of two ligands simultaneously 2

A simple model for inhibitor binding 2

A simple model for TPX2 binding 3

Induced fit and conformational selection following conformational interconversion 4

Binding reactions in full thermodynamic cycle 4

Experimental methods **6**

Buffers 6

Inhibitors 6

Protein expression 6

TMR labelling of surface cysteine residues of Aurora-A 6

Mechanism of dye quenching 7

Kinase activity assay 7

Functionalization and PEGylation of glass slides and coverslips 8

Construction of flow cell 8

Immobilization of Aurora-A 8

TIRF imaging and single molecule data analysis 9

Assignment of intensity changes to conformational change 10

Data fitting 10

Full references **12**

Supplementary table **14**

Supplementary figures **15**

Reaction scheme modelling

Calculation of the expected population of inactive T-loop conformation in the presence of two ligands simultaneously

In order to determine whether the binding of activator and inhibitor ligands is mutually exclusive, we can simplify the system under consideration to the following scheme:

$$AurA\text{-}MLN \underset{+MLN}{\rightleftharpoons} AurA \overset{+TPX2}{\rightleftharpoons} AurA\text{-}TPX2$$

Expressions for the dissociation constants of TPX2 and MLN8054 are as follows:

$$K_{d_{TPX2}} = \frac{[TPX2][AurA]}{[AurA\text{-}TPX2]} \tag{S1}$$

$$K_{d_{MLN}} = \frac{[MLN][AurA]}{[AurA\text{-}MLN]} \tag{S2}$$

where [AurA] is the concentration of free Aurora-A, [TPX2] the concentration of free TPX2, [MLN] the concentration of free MLN8054, and [AurA-TPX2] and [AurA-MLN] are the concentrations of Aurora-A bound to TPX2 and MLN8054 respectively.

Rearranging (S1) for [AurA] and substituting into (S2) gives the following expression:

$$\frac{[AurA\text{-}TPX2]}{[AurA\text{-}MLN]} = \frac{K_{d_{MLN}}}{K_{d_{TPX2}}} \cdot \frac{[TPX2]}{[MLN]} \approx \frac{K_{d_{MLN}}}{K_{d_{TPX2}}} \cdot \frac{[TPX2_{total}]}{[MLN_{total}]} \tag{S3}$$

Since our experiments were carried out under conditions where both ligands are present in vast excess, [TPX2] and [MLN] can be approximated by the total concentration of each ligand. This enables a numerical value for the ratio of ligated Aurora-A molecules to be calculated as 0.015. Weighting this value by the measured percentages of inactive T-loop conformation for each ligand individually gives the expected population of the inactive T-loop conformation in a binding model where each Aurora-A molecule binds only one ligand at a time.

A simple model for inhibitor binding

$$Active \rightleftharpoons Inactive \overset{+inh}{\rightleftharpoons} Inactive\text{-}inh \tag{1}$$

We can write down expressions for the dissociation constant of inhibitor from the inactive T-loop conformation and for the overall equilibrium constant as follows:

$$K_{d,inh,inactive} = \frac{[inh][Inactive]}{[Inactive\text{-}inh]} \quad \textbf{(S4)}$$

$$K_{eq} = \frac{[Inactive]+[Inactive\text{-}inh]}{[Active]} = K_{eq}^{*}\left(1+\frac{[inh]}{K_{d,inh,inactive}}\right) \quad \textbf{(S5)}$$

where K_{eq} is the equilibrium constant for the complete system, K_{eq}* is the equilibrium constant [Inactive]/[Active] and $K_{d,inh,inactive}$ is the dissociation constant for inhibitor from the inactive T-loop conformation.

The measured dissociation constant, $K_{d,inh}$, can be expressed as

$$K_{d,inh} = \frac{[inh]([Inactive]+[Active])}{[Inactive\text{-}inh]} = \frac{K_{d,inh,inactive}\left(1+K_{eq}^{*}\right)}{K_{eq}^{*}} \quad \textbf{(S6)}$$

and rearranged to give an expression for $K_{d,inh,inactive}$.

Using experimental values of K_{eq}* = 0.3 (Table I, apo conditions), $K_{d,MLN}$=0.4 nM and [MLN8054] = 10 µM we obtain calculated values of $K_{d,MLN,inactive}$ = 0.1 nM and K_{eq} = 30,000 – vastly different to the experimental value K_{eq} = 0.7.

A simple model for TPX2 binding

$$Active\text{-}TPX2 \underset{+TPX2}{\rightleftharpoons} Active \rightleftharpoons Inactive$$

From a scheme equivalent to (1) in the main text, where TPX2 binds the active T-loop conformation and active T-loop remains in equilibrium with inactive T-loop, we can derive expressions as follows:

$$K_{d,TPX2} = K_{d,TPX2,active}\left(1+K_{eq}^{*}\right) \quad \textbf{(S7)}$$

$$K_{eq} = \frac{K_{eq}^{*}}{1+\frac{[TPX2]}{K_{d,TPX2,down}}} \quad \textbf{(S8)}$$

Using experimental values of K_{eq}* = 0.3, $K_{d,TPX2}$ = 10 nM and [TPX2] = 5 µM, gives calculated values of $K_{d,TPX2}$ = 7 nM and K_{eq} = 0.0004 – again vastly different to the experimental value K_{eq} = 0.2.

Induced fit (2) and conformational selection (3) following conformational interconversion

$$Active \rightleftharpoons Inactive \xrightleftharpoons{+inh} Inactive\text{-}inh \rightleftharpoons Inactive^{*}\text{-}inh \quad \textbf{(2)}$$

$$Active \rightleftharpoons Inactive \rightleftharpoons Inactive^{*} \xrightleftharpoons{+inh} Inactive^{*}\text{-}inh \quad \textbf{(3)}$$

Expressions for K_d and for K_{eq} can be written down / derived in a similar manner to equations (S4), (S5) and (S6). Experimental values can then be compared with those calculated.

Binding reactions in full thermodynamic cycle

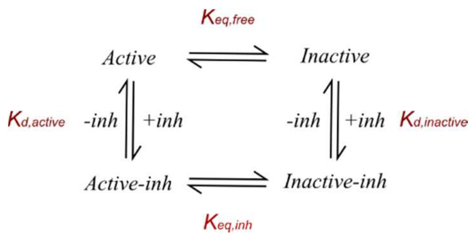

(4)

The conformational equilibrium constants can be defined as follows:

$$K_{eq,free} = \frac{[Inactive]}{[Active]} \quad \textbf{(S9)}$$

$$K_{eq,inh} = \frac{[Inactive\text{-}inh]}{[Active\text{-}inh]} \quad \textbf{(S10)}$$

where [Inactive] and [Active] are the concentrations of unbound kinase in the indicated T-loop conformations, and [Inactive-inh] and [Active-inh] are the concentrations of inhibitor-bound kinase in inactive and active T-loop conformations respectively.

Likewise, the dissociations constants for inhibitor dissociation from inactive and active T-loop conformations are:

$$K_{d,inactive} = \frac{[inh][Inactive]}{[Inactive\text{-}inh]} \quad \textbf{(S11)}$$

$$K_{d,active} = \frac{[inh][Active]}{[Active\text{-}inh]} \quad \textbf{(S12)}$$

The measured dissociation constant, K_d, is

$$K_d = \frac{[inh][enz]}{[enz\text{-}inh]} = \frac{[inh]([Inactive]+[Active])}{[Inactive\text{-}inh]+[Active\text{-}inh]} \quad \textbf{(S13)}$$

and the experimental equilibrium constant, K_{eq}, is

$$K_{eq} = \frac{[Inactive]+[Inactive-inh]}{[Active]+[Active-inh]} \quad \textbf{(S14)}$$

Substituting into (S13) and (S14) from (S9), (S10) and (S12) gives

$$K_d = \frac{K_{d,active}\left(1+K_{eq,free}\right)}{1+K_{eq,inh}} \quad \textbf{(S15)}$$

$$K_{eq} = \frac{K_{eq,free}K_{d,active}+K_{eq,inh}[inh]}{K_{d,active}+[inh]} \quad \textbf{(S16)}$$

which can be rearranged and substituted once more to express $K_{eq,inh}$ in terms of experimental parameters:

$$K_{eq,inh} = \frac{K_{eq}[inh]\left(1+K_{eq,free}\right)+K_d\left(K_{eq}-K_{eq,free}\right)}{[inh]\left(1+K_{eq,free}\right)-K_d\left(K_{eq}-K_{eq,free}\right)} \quad \textbf{(S17)}$$

Equation (S11) can also be rearranged and substituted into from (S9), (S10) and (S15) to give

$$\frac{K_{d,inactive}}{K_{d,active}} = \frac{K_{eq,free}}{K_{eq,inh}} \quad \textbf{(S18)}$$

Rearranging equations (S15) and (S18) gives expressions for $K_{d,active}$ and $K_{d,inactive}$, enabling both quantities to be calculated. Similar relationships hold when the enzyme is bound to TPX2 throughout.

Experimental methods

Buffers

Labelling buffer: 50 mM Tris-HCl pH 7.5, 200 mM NaCl, 5 mM $MgCl_2$, 10% glycerol

Kinase buffer: 50 mM Tris-HCl pH7.5, 200 mM NaCl, 5 mM $MgCl_2$, 10% glycerol, 1 mM DTT

Imaging buffer: 0.3 mg/mL BSA, 50 mM Tris-HCl pH7.5, 200 mM NaCl, 5 mM $MgCl_2$, 10% glycerol, 5 mM protocatechuic acid, 0.1 µM protocatechuate 3,4-dioxygenase, 1% DMSO and 5 mM Trolox

Inhibitors

MLN8054 was purchased from Selleck Chemicals. CD532 was prepared according to the literature method.[5d]

Protein expression

S283C, K224C and S283C/K224C point mutations were generated in His-tagged C290A/C393A Aurora-A kinase domain (residues 122-403) using QuikChange. Like wildtype Aurora-A, C290A/C393A autophosphorylates in *E.coli*, and is purified pre-phosphorylated on Thr288.[7a] An unstructured linker of 24 residues separates the His-tag from the N-terminus of the kinase domain and ensures free rotation of the immobilized kinase in solution. The linker sequence is as follows: MHHHHHHSSGLVPRGSGMKETAAAKFEENLYFQGA. All proteins were expressed in *E.coli* and purified as previously described.[4b, 13]

TMR labelling of surface cysteine residues of Aurora-A

A 200 µL sample of Aurora-A was buffer exchanged into labelling buffer using a 5 mL desalting column. 10 mM of 5'-tetramethylrhodamine iodoacetemide (TMRIA) in DMSO was added to the eluent protein solution at a molar ratio of 1:15 protein:TMRIA for single mutant Aurora-A samples (K224C, S283C) and a ratio of 1:20 protein:TMRIA for double mutant Aurora-A (S283C/K224C) and incubated at 4ºC on rollers in the dark overnight. The reaction was quenched with 1 M DTT at a ratio of at least 10:1 DTT:TMRIA, the sample concentrated and unreacted TMRIA removed using a 10 mL desalting column equilibrated with labelling buffer supplemented with 1mM DTT. Labelling efficiency was determined by the ratio of total protein to total dye (total protein and dye quantified by absorbance at

280 nm and 514 nm respectively) assuming random labelling. Samples were frozen at -80ºC for future use.

The labelling efficiency of K224C/S283C was 120% labelling overall. Assuming 60% labelling at each site, this results in 36% double labelled, 48% single labelled and 16% unlabelled protein.

Mechanism of dye quenching

Like previous work[8], our assay uses the self-quenching of TMR fluorescence to report on short distance changes in the conformation of the protein kinase activation loop. There is very little mechanistic information on this phenomenon in the literature, and the kinetics of TMR association / dissociation are uncharacterized. TMR can form a dimer in protein crystals[14] (static quenching) and the K_d determined spectroscopically for a dimer of TMR-labelled rabbit myosin fragments is 137 μM.[15] It is also possible that there is also a mechanistic contribution from dynamic quenching, similar to that determined for the dye-quencher pair MR121-tryptophan.[16] Formally, the kinetics of TMR dissociation may thus provide an upper to the rate constants that can be determined in our measurements.

Nevertheless, rate constants of ~3 s^{-1} (*ie* slightly faster than those reported here) have previously been reported for the ADP-sensing protein ParM using TMR self-quenching.[8] These were found to be consistent with independent stopped flow data, giving us confidence that TMR self-quenching provides a reliable probe of kinetic processes on this timescale.

Kinase activity assay

Aurora-A kinase activity was determined using the ADP-Glo^{TM} Kinase Assay kit (Promega), following the manufacturer's instructions. Briefly, 25 µl kinase reactions were carried out in 96-well plates for 1 hour at room temperature in Kinase buffer. The reaction was stopped and the remaining ATP depleted by addition of 25 µl ADP-Glo reagent for 40 minutes. Kinase detection reagent (50 µl) was then added to convert ADP to ATP and allow the luciferin/luciferase reaction to take place. Plates were incubated at room temperature for 1 hour, and luminescence was detected using a BioTek luminescence plate reader. The amount of ADP produced was determined using a standard curve, run in each plate alongside the other assays. Peptide K_m experiments were carried out using a twofold serial dilution from 2 mM Kemptide in buffer.

Functionalization and PEGylation of glass slides and coverslips

Aurora-A molecules were bound to the surface of functionalized coverslips and glass slides for fluorescence microscopy. Two 1.5 mm holes were drilled in the microscope slide approximately 5 mm apart. The coverslips and slides were super-cleaned with separate washes of Alconox, ethanol and MiliQ water solutions. The coverslips and slides were then functionalized with an amino-group through incubating with 1 in100 dilution of 3-aminopropyltheithoxysilane (sold as Vectabond) in methanol / 5% acetic acid for 20 minutes. Glass surfaces were PEGylated by incubating with 28.5 mM PEG-SVA:biotin-PEG-SVA solution (7:1 ratio) in 10 mM $NaHCO_3$ solution for 2 hours, followed by washing with MiliQ water and drying with N_2 gas.

Construction of flow cell

Functionalized slides and coverslips were used to construct flow cells as follows. 1.5 mm diameter tubing (Agilent Technologies, UK) was glued into each 1.5 mm hole with epoxy resin glue and cut flush on the functionalized side with a razor. Two self-adhesive 10 x 10 mm, 25 µL Gene Frames (Thermofisher Scientific, UK) were stacked around the inlet and outlet holes on the functionalized side of the slide and a functionalized coverslip glued on top using epoxy resin (functionalized side towards the slide) (Figure S4b). Solutions were injected along the inlet tubing at a low (<0.25 mL/min) flow rate.

Immobilization of Aurora-A

Aurora-A was tethered to the glass coverslip inside the flow cell using a biotinylated PEG–neutravidin–biotinylated anti-His–His-tagged protein strategy as follows [8, 17]: i) Flow cells were first incubated in NeutrAvadin (0.1 mg/mL) in 50 mM Tris-HCl pH7.5, 50 mM NaCl for one hour to bind to the covalently bonded PEG-biotin conjugate. Excess NeutrAvidin was eluted with a ~1.5 mL wash of 50 mM Tris-HCl pH 7.5, 50 mM NaCl solution. ii) A biotinylated anti-His antibody (Qiagen, UK) at 1 in 1000 dilution in 30 µg/mL BSA, 50 mM NaCl, 50 mM Tris-HCl pH 7.5 was incubated for 10 minutes to conjugate to the NeutrAvadin. Excess antibody was eluted with a 1.5 mL wash of 50 mM Tris-HCl pH 7.5, 50 mM NaCl solution. iii) In low light conditions, a solution of labelled Aurora-A (<10 nM) in labelling buffer supplemented with 0.3 mg/mL BSA was injected in the flow cell and equilibrated for 10 min, before being washed out with ~1.5 mL imaging buffer. We expect any non-specifically bound molecules to be removed by this wash, or to have very short residence times within the illumination of

the evanescent wave as they exchange with bulk solvent in the flow cell. For measurements with inhibitors, activators or substrates, 0.5 mL of solution containing the target binding molecule in imaging buffer (made up to a final concentration of 1% DMSO as necessary) was injected and allowed to incubate in darkness for 10 minutes. CD532 and MLN8054 were dissolved in DMSO to a concentration of 1 mM and added to the final solution at a ratio of 1:100.

TIRF imaging and single molecule data analysis

Samples were illuminated in TIRF using a ~2.0 mW 514 nm laser. Filters were ZET514 (excitation; Chroma), ZT514rdc (dichroic; Chroma) and both HQ525 longpass (emission; Chroma) and 595RDF60 (emission; Omega). Fluorescence was captured by a CoolView EM 1000 camera using an 80 ms/frame capture speed and 2x2 pixel binning with 500 frames per video.

The captured tiff video of the fluorescent molecules was processed with custom written IDL code. In summary, the complete tiff stack was used to generate a time-averaged image. Background intensity values were subtracted from this. High-intensity fluorescent molecules were identified by scanning for pixels above an intensity threshold and recording the peak location if the surrounding pixels were below one standard deviation. In the video stack, the intensity of the 8x8 pixel area surrounding each peak was enhanced by multiplying by the weighted values of a 3D Gaussian curve centered on the high intensity pixel, and the sum intensity of the curve in each frame was collected as intensity vs time.

The fluorescence intensity over time of each molecule was visualized using custom Matlab code. The fluorescence intensity trace was smoothed with a 3-point moving average, and double-labelled fluorescent molecules were identified manually as traces ending with a clear two-step photobleaching event (Figure 1f). Approximately 5% of traces identified by the IDL code were classified as double-labelled fluorescent molecules. The thresholding for trace identification was deliberately kept low to avoid missing molecules predominantly in the inactive conformation (trace identification was based on average intensity over the acquisition) and traces classified as not double-labelled fluorescent molecules included false-positives, data where the initial intensity was background or which dropped to background without two-step photobleaching (potential non-specific binders transiently associating with or diffusing on the surface), aggregates and single-labelled protein traces.

Double-labelled fluorescence intensity traces were isolated, and at least 100 molecules (average of 121 frames per molecule) combined to create each intensity histogram (each bar within the histogram indicates the total number of frames for which a particular fluorescence intensity was observed). Histograms were normalized to a total area of 1 for easy visual comparison.

We discovered that some particularly low-intensity frames of quenched events were omitted from our histograms due to the background subtraction (negative intensities). Such frames were infrequent stochastic variations in intensity rather than complete events, but omission of these values led to poor definition of the left hand side of the low intensity histogram peak, partly because the function must pass through the origin. This propagated into poor fits of the double log normal function which could be overcome by a small x-axis translation to make all values positive. We modelled the effect of such a translation on idealized data sets and determined that unnecessarily translating well-defined data introduced an eventual error on reported curve areas of ~2%, equivalent to the fitting error on our experimental measurements. We additionally determined that failure to include these low intensity values by only fitting positive background-subtracted intensities was likely to introduce errors of ~3% (Figure S7). We thus applied an x-axis translation to all our data by adding 300 a.u. to all fluorescent intensities. This value was chosen as the smallest number required to make all intensity bins positive.

Assignment of intensity changes to conformational change

Prior to two-step photobleaching, double-labelled molecules exhibited a single high fluorescence intensity and transiently entered a low intensity, quenched, state. The quenched state was never observed after the first photobleaching event, nor in control measurements on single-labelled protein (K224C and S283C), and thus was not due to photophysical dye blinking.[18] Photobleaching occurring directly from the quenched state resulted in an increase in fluorescence to that expected for a single dye molecule. We therefore assigned changes in our fluorescence signal to changes in the position of the Aurora-A T-loop: high fluorescence indicating an active T-loop, quenched fluorescence an inactive T-loop.

Data fitting

Consistent with observations elsewhere in the literature [19], fluorescence intensity distributions for single-labelled control protein (Figure S5a&b) were fit to a log normal distribution (S19) and for

double-labelled protein (Figure 2) to the sum of two log normal distributions (S20) in Prism using the equations below:

$$y = \frac{A}{x} e^{\frac{-\left(\ln x - \text{mode} - \sigma^2\right)^2}{2\sigma^2}} \qquad \textbf{(S19)}$$

$$y = \frac{A_1}{x} e^{\frac{-\left(\ln x - \text{mode}_1 - \sigma_1^2\right)_1}{2\sigma_1^2}} + \frac{A_2}{x} e^{\frac{-\left(\ln x - \text{mode}_2 - \sigma_2^2\right)_2}{2\sigma_2^2}} \qquad \textbf{(S20)}$$

The mode (peak) of each distribution is e^{mode} and the area under each curve is $A\sigma\sqrt{2\pi}$.

The dwell time histogram (Figure 2b), which is constructed from the duration of each quenching event, was fit to a single exponential decay using Prism.

Full references

[1] a) P. Cohen, *Nature Reviews Drug Discovery* **2002**, *1*, 309-315; b) P. J. Barnes, *Nat Rev Drug Discov* **2013**, *12*, 543-559; c) P. Wu, T. E. Nielsen, M. H. Clausen, *Trends Pharmacol Sci* **2015**, *36*, 422-439.

[2] a) Y. Meng, B. Roux, *J Mol Biol* **2014**, *426*, 423-435; b) L. N. Johnson, M. E. M. Noble, D. J. Owen, *Cell* **1996**, *85*, 149-158.

[3] a) M. Vogtherr, K. Saxena, S. Hoelder, S. Grimme, M. Betz, U. Schieborr, B. Pescatore, M. Robin, L. Delarbre, T. Langer, K. U. Wendt, H. Schwalbe, *Angewandte Chemie-International Edition* **2006**, *45*, 993-997; b) M. A. Morando, G. Saladino, N. D'Amelio, E. Pucheta-Martinez, S. Lovera, M. Lelli, B. Lopez-Mendez, M. Marenchino, R. Campos-Olivas, F. L. Gervasio, *Sci Rep* **2016**, *6*, 24439.

[4] a) R. Bayliss, T. Sardon, I. Vernos, E. Conti, *Molecular Cell* **2003**, *12*, 851-862; b) C. A. Dodson, R. Bayliss, *Journal of Biological Chemistry* **2012**, *287*, 1150-1157; c) P. A. Eyers, J. L. Maller, *J Biol Chem* **2004**, *279*, 9008-9015; d) K. Anderson, J. Yang, K. Koretke, K. Nurse, A. Calamari, R. B. Kirkpatrick, D. Patrick, D. Silva, P. J. Tummino, R. A. Copeland, Z. Lai, *Biochemistry* **2007**, *46*, 10287-10295.

[5] a) V. Bavetsias, A. Faisal, S. Crumpler, N. Brown, M. Kosmopoulou, A. Joshi, B. Atrash, Y. Perez-Fuertes, J. A. Schmitt, K. J. Boxall, R. Burke, C. Sun, S. Avery, K. Bush, A. Henley, F. I. Raynaud, P. Workman, R. Bayliss, S. Linardopoulos, J. Blagg, *J Med Chem* **2013**, *56*, 9122-9135; b) C. A. Dodson, M. Kosmopoulou, M. W. Richards, B. Atrash, V. Bavetsias, J. Blagg, R. Bayliss, *Biochemical Journal* **2010**, *427*, 19-28; c) S. Geuns-Meyer, V. J. Cee, H. L. Deak, B. Du, B. L. Hodous, H. N. Nguyen, P. R. Olivieri, L. B. Schenkel, K. R. Vaida, P. Andrews, A. Bak, X. Be, P. J. Beltran, T. L. Bush, M. K. Chaves, G. Chung, Y. Dai, P. Eden, K. Hanestad, L. Huang, M. H. Lin, J. Tang, B. Ziegler, R. Radinsky, R. Kendall, V. F. Patel, M. Payton, *J Med Chem* **2015**, *58*, 5189-5207; d) W. C. Gustafson, J. G. Meyerowitz, E. A. Nekritz, J. Chen, C. Benes, E. Charron, E. F. Simonds, R. Seeger, K. K. Matthay, N. T. Hertz, M. Eilers, K. M. Shokat, W. A. Weiss, *Cancer Cell* **2014**, *26*, 414-427; e) M. Kollareddy, D. Zheleva, P. Dzubak, P. S. Brahmkshatriya, M. Lepsik, M. Hajduch, *Invest New Drugs* **2012**, *30*, 2411-2432; f) H. R. Lawrence, M. P. Martin, Y. Luo, R. Pireddu, H. Yang, H. Gevariya, S. Ozcan, J. Y. Zhu, R. Kendig, M. Rodriguez, R. Elias, J. Q. Cheng, S. M. Sebti, E. Schonbrunn, N. J. Lawrence, *J Med Chem* **2012**, *55*, 7392-7416; g) M. G. Manfredi, J. A. Ecsedy, K. A. Meetze, S. K. Balani, O. Burenkova, W. Chen, K. M. Galvin, K. M. Hoar, J. J. Huck, P. J. LeRoy, E. T. Ray, T. B. Sells, B. Stringer, S. G. Stroud, T. J. Vos, G. S. Weatherhead, D. R. Wysong, M. K. Zhang, J. B. Bolen, C. F. Claiborne, *Proceedings of the National Academy of Sciences of the United States of America* **2007**, *104*, 4106-4111.

[6] a) M. W. Richards, S. G. Burgess, E. Poon, A. Carstensen, M. Eilers, L. Chesler, R. Bayliss, *Proc Natl Acad Sci U S A* **2016**; b) M. Brockmann, E. Poon, T. Berry, A. Carstensen, H. E. Deubzer, L. Rycak, Y. Jamin, K. Thway, S. P. Robinson, F. Roels, O. Witt, M. Fischer, L. Chesler, M. Eilers, *Cancer Cell* **2013**, *24*, 75-89; c) S. G. DuBois, A. Marachelian, E. Fox, R. A. Kudgus, J. M. Reid, S. Groshen, J. Malvar, R. Bagatell, L. Wagner, J. M. Maris, R. Hawkins, J. Courtier, H. Lai, F. Goodarzian, H. Shimada, S. Czarnecki, D. Tsao-Wei, K. K. Matthay, Y. P. Mosse, *J Clin Oncol* **2016**, *34*, 1368-1375.

[7] a) S. G. Burgess, R. Bayliss, *Acta Crystallographica Section F-Structural Biology Communications* **2015**, *71*, 315-319; b) F. C. Rowan, M. Richards, R. A. Bibby, A. Thompson, R. Bayliss, J. Blagg, *Acs Chemical Biology* **2013**, *8*, 2184-2191.

[8] R. B. Zhou, S. Kunzelmann, M. R. Webb, T. Ha, *Nano Letters* **2011**, *11*, 5482-5488.

[9] T. Ha, P. Tinnefeld, in *Annual Review of Physical Chemistry, Vol 63, Vol. 63* (Eds.: M. A. Johnson, T. J. Martinez), Annual Reviews, Palo Alto, **2012**, pp. 595-617.

[10] a) R. V. Agafonov, C. Wilson, R. Otten, V. Buosi, D. Kern, *Nature Structural & Molecular Biology* **2014**, *21*, 848-853; b) C. Wilson, R. V. Agafonov, M. Hoemberger, S. Kutter, A. Zorba, J. Halpin, V. Buosi, R. Otten, D. Waterman, D. L. Theobald, D. Kern, *Science* **2015**, *347*, 882-

886; c) A. C. Dar, M. S. Lopez, K. M. Shokat, *Chem Biol* **2008**, *15*, 1015-1022; d) M. A. Seeliger, P. Ranjitkar, C. Kasap, Y. Shan, D. E. Shaw, N. P. Shah, J. Kuriyan, D. J. Maly, *Cancer Res* **2009**, *69*, 2384-2392.
[11] a) A. Aleksandrov, T. Simonson, *J Biol Chem* **2010**, *285*, 13807-13815; b) Y. L. Lin, Y. Meng, W. Jiang, B. Roux, *Proc Natl Acad Sci U S A* **2013**, *110*, 1664-1669; c) S. Lovera, L. Sutto, R. Boubeva, L. Scapozza, N. Dolker, F. L. Gervasio, *J Am Chem Soc* **2012**, *134*, 2496-2499.
[12] Y. Xiao, T. Lee, M. P. Latham, L. R. Warner, A. Tanimoto, A. Pardi, N. G. Ahn, *Proc Natl Acad Sci U S A* **2014**, *111*, 2506-2511.
[13] C. A. Dodson, *Methods Mol Biol* **2017**, *1586*, 251-264.
[14] a) A. B. Edmundson, K. R. Ely, J. N. Herron, *Mol Immunol* **1984**, *21*, 561-576; b) M. J. Blackman, J. E. Corrie, J. C. Croney, G. Kelly, J. F. Eccleston, D. M. Jameson, *Biochemistry* **2002**, *41*, 12244-12252.
[15] K. Ajtai, P. J. Ilich, A. Ringler, S. S. Sedarous, D. J. Toft, T. P. Burghardt, *Biochemistry* **1992**, *31*, 12431-12440.
[16] S. Doose, H. Neuweiler, M. Sauer, *Chemphyschem* **2005**, *6*, 2277-2285.
[17] R. Lamichhane, A. Solem, W. Black, D. Rueda, *Methods* **2010**, *52*, 192-200.
[18] J. Schuster, J. Brabandt, C. von Borczyskowski, *Journal of Luminescence* **2007**, *127*, 224-229.
[19] S. A. Mutch, B. S. Fujimoto, C. L. Kuyper, J. S. Kuo, S. M. Bajjalieh, D. T. Chiu, *Biophys J* **2007**, *92*, 2926-2943.
[20] N. M. Heron, M. Anderson, D. P. Blowers, J. Breed, J. M. Eden, S. Green, G. B. Hill, T. Johnson, F. H. Jung, H. H. McMiken, A. A. Mortlock, A. D. Pannifer, R. A. Pauptit, J. Pink, N. J. Roberts, S. Rowsell, *Bioorg Med Chem Lett* **2006**, *16*, 1320-1323.

Supplementary table

Table SI: Fitted parameters from intensity histograms

Histogram	Width parameter[b]	Calculated mode[b]
S283C[a]	0.530 ± 0.010	1199 ± 15
K224C[a]	0.374 ± 0.008	1749 ± 15
S283C/K224C	0.371 ± 0.008	2939 ± 28
S283C + MLN8054	0.559 ± 0.008	1265 ± 12
K224C + MLN8054	0.477 ± 0.007	1201 ± 9
S283C + CD532	0.395 ± 0.005	1355 ± 9

[a]The difference of ~500 a.u. between the modes of S283C and K224C is likely to be due to the differing molecular environments of each dye molecule within the structure of the protein.
[b]Error is fitting error (width) or error propagated from this (calculated mode).

Supplementary figures

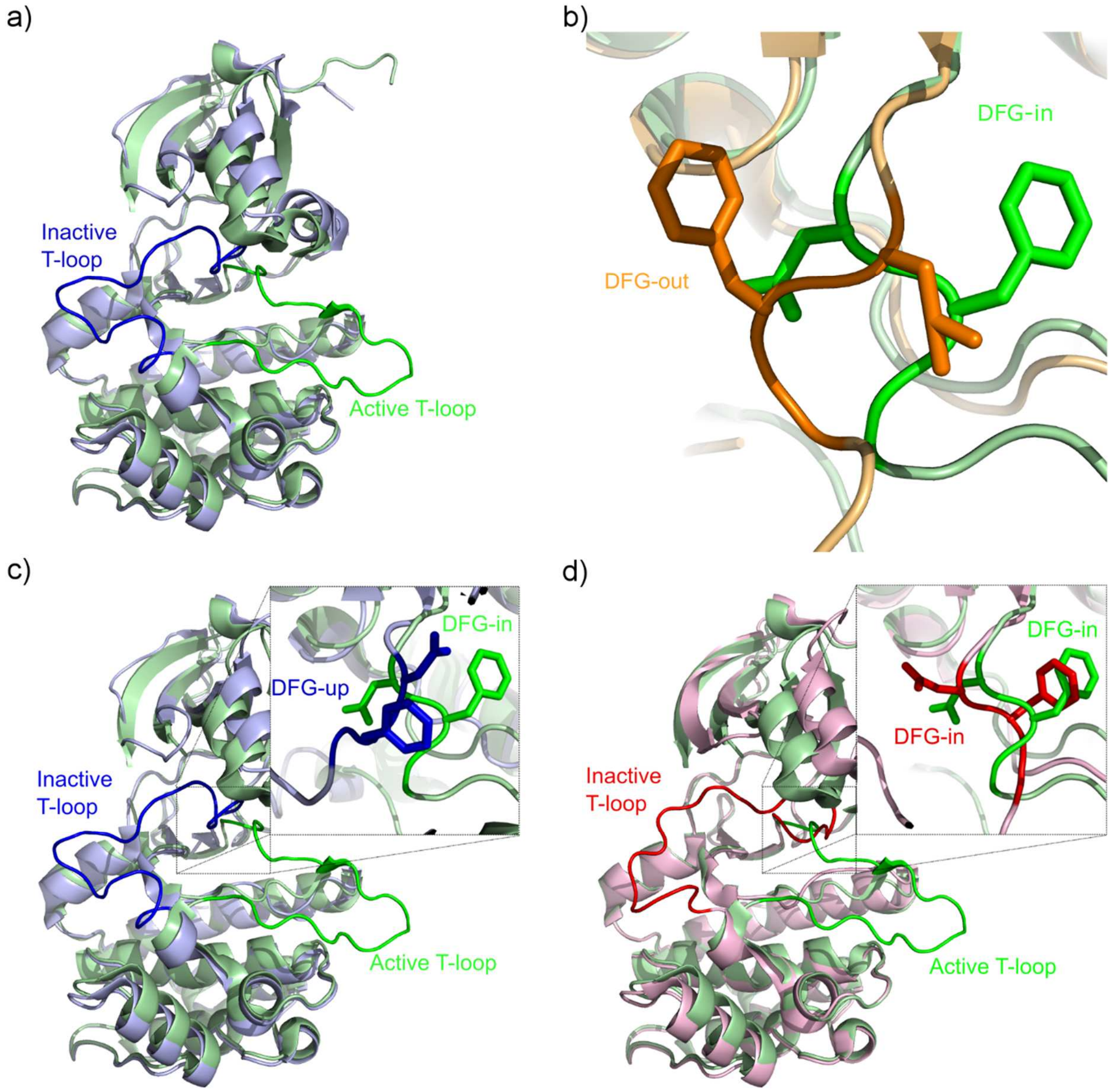

Figure S1: Naming conventions for different conformations of Aurora-A. a) Active T-loop (active kinase; green; PDB 1OL5) and inactive T-loop (inactive kinase; blue; PDB 2WTV) conformations. b) DFG-in (active kinase bound to ADP; green; PDB 1OL5) and DFG-out (inactive kinase bound to compound 13 [20]; orange, PDB 2C6E) conformations. c) Active T-loop (active kinase bound to ADP; green; PDB 1OL5) and inactive T-loop conformations (inactive kinase bound to MLN8054; blue; PDB 2WTV), and the non-conventional conformation of the DFG motif of aurora-A kinase bound to MLN8054 (blue, inset) in which the aspartic acid residue is orientated in the same direction as the phenylalanine residue (DFG-up), compared to the DFG-in conformation of aurora-A binding TPX2 (green). d) The inactive T-loop (aurora-A kinase bound to CD532 inhibitor; red; PDB 4J8M) and active T-loop (active kinase bound to ADP; green; PDB 1OL5) conformations, and the DFG-in (red; inset) conformation of the DFG motif of aurora-A bound to CD532, which is aligned in the same conformation as that of active aurora-A kinase (green).

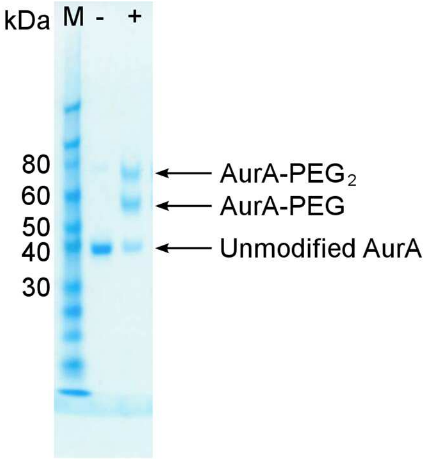

Figure S2: PEG labelling reaction. M – marker (sizes indicated on left); '-' – Aurora-A K224C/S283C control; '+' – Aurora-A K224C/S283C reacted with 10x molar excess of PEG maleimide. A maximum of two AurA-PEG bands are observed, indicating that only two thiol groups are available for reaction with maleimide.

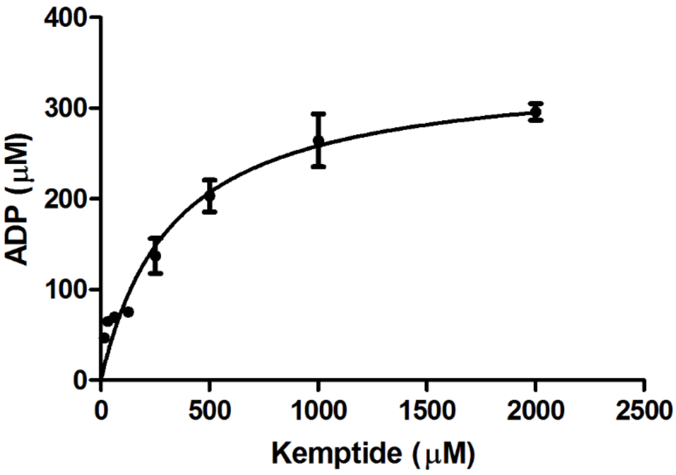

Figure S3: Peptide Km for double labelled Aurora-A. Activity shown as [ADP] produced over the course of a 1hr reaction.

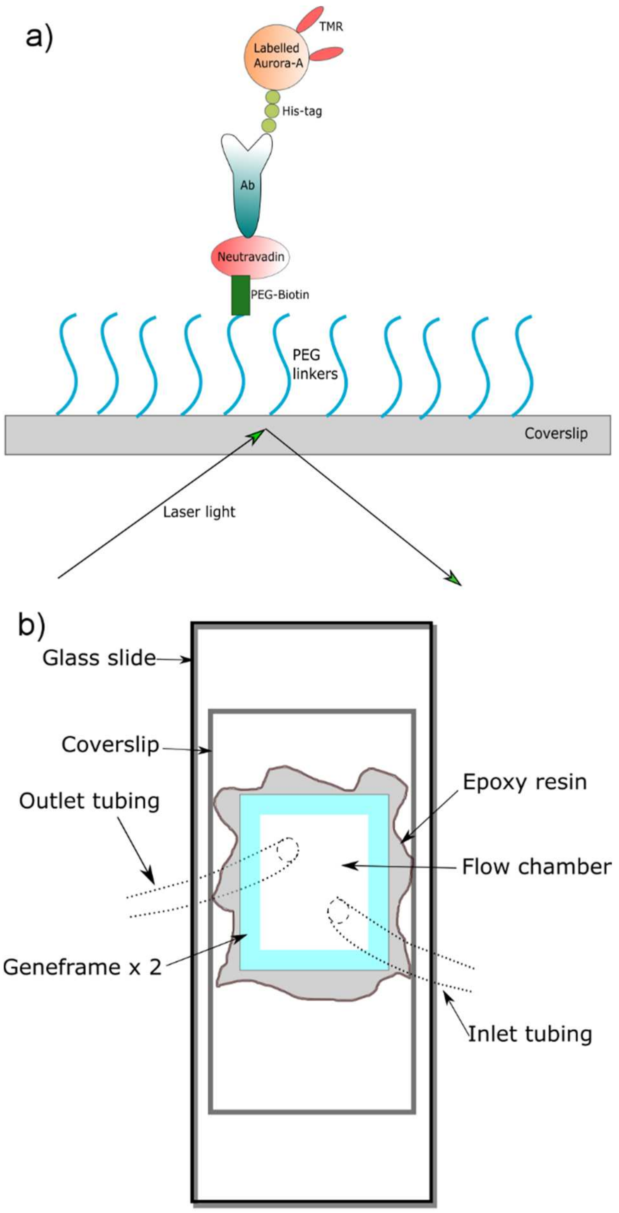

Figure S4: Flow cell and tethering strategy. a) Cartoon representation of the conjugate chain of linkers anchoring Aurora-A to the internal surface of a coverslip, and the angle of the incident laser light. b) Construction of the flow cell used to image the fluorescent molecules. In this image, the glass slide is at the rear and the coverslip at the front.

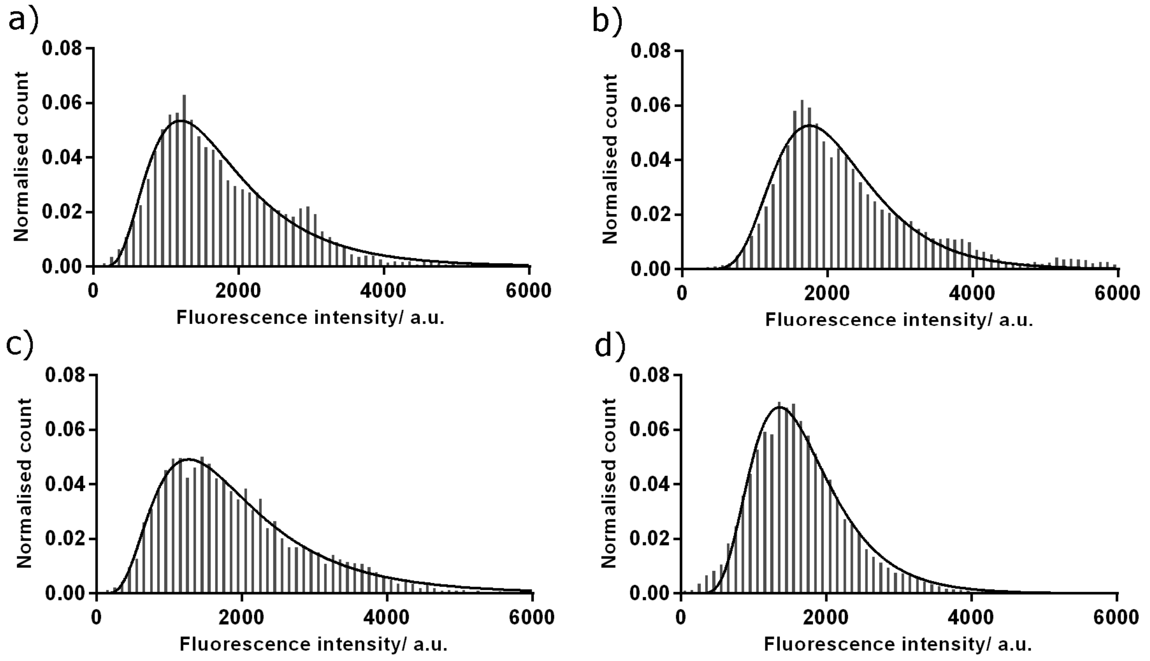

Figure S5: Fluorescence intensity histograms for single labelled protein a) S283C; b) K224C; c) S283C in the presence of 10 µM MLN8054 d) S283C in the presence of 10 µM CD532.

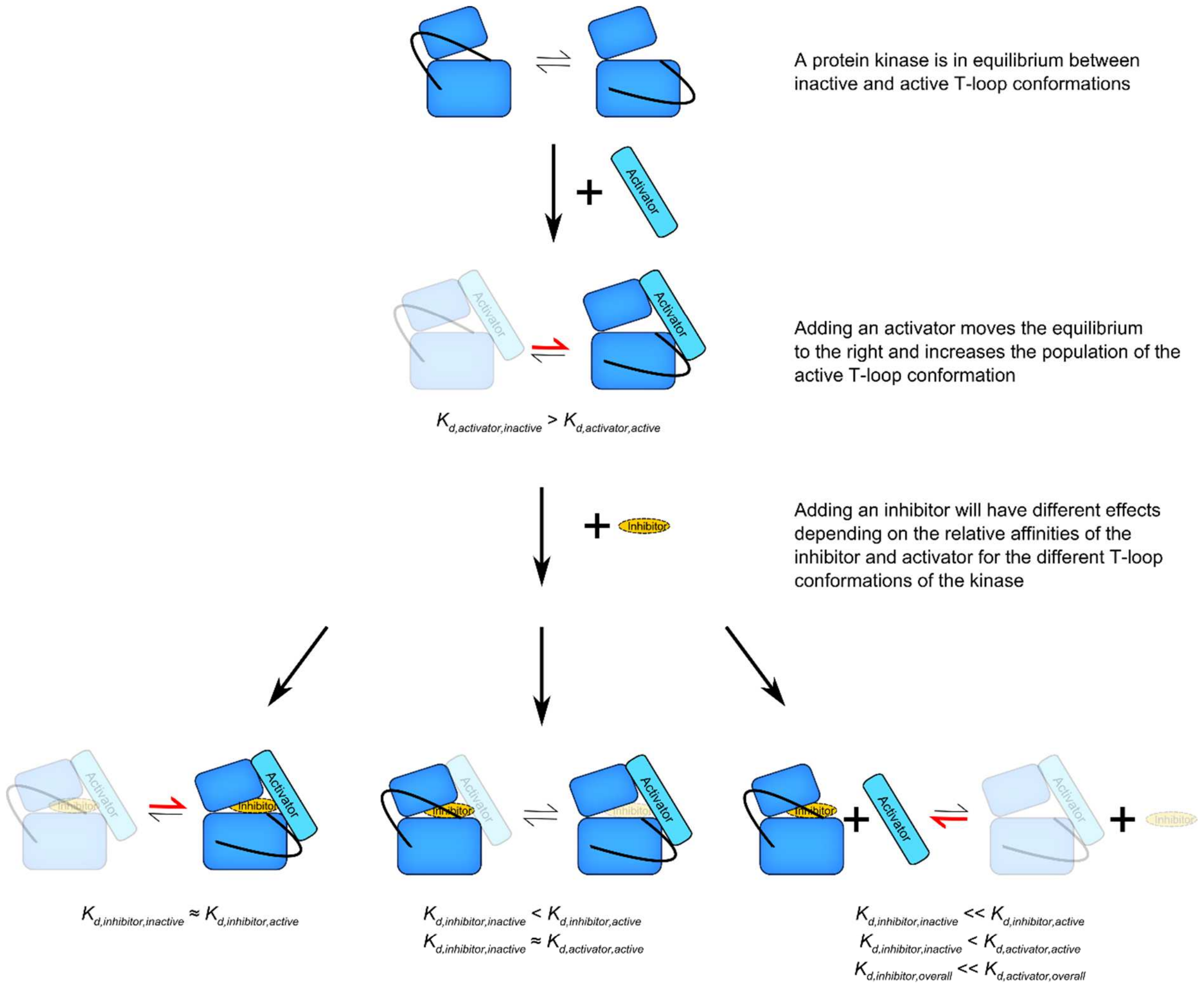

The inhibitor has a similar affinity for active and inactive conformations of the kinase. It does not move the position of equilibrium and forms a triple complex in active and inactive conformations.

The overall population of active and inactive T-loop conformations is as for kinase with activator alone.

The inhibitor binds more strongly to the inactive T-loop conformation. It binds the inactive T-loop conformation about as strongly as the activator binds the active T-loop conformation.

The inhibitor increases the population of the inactive T-loop conformation. Some of these kinase molecules will be bound to inhibitor alone, others will be bound to inhibitor and activator in a triple complex (depending on the relative affinities of the activator and inhibitor for the inactive T-loop conformation).

There is still a population of kinase in the active T-loop conformation. The majority of this is bound to activator, some is also bound to inhibitor in a triple complex.

There may be a small population of free activator in solution depending on its overall affinity for the kinase.

The inhibitor binds very strongly to the inactive T-loop conformation and barely binds the active conformation at all. It binds the inactive T-loop conformation more strongly than the activator binds the active conformation. It thus moves the position of equilibrium to the left and increases the population of the inactive T-loop conformation.

The activator has a weak affinity for the inactive T-loop conformation and so only a small proportion of activator binds the kinase. The weaker the affinity of the activator for the inactive conformation, the less triple complex is formed in the inactive T-loop conformation and the greater the population of free activator in solution.

Depending on the relative affinities of the inhibitor for the inactive T-loop conformation and the activator for the active T-loop conformation, a small population of activator-bound kinase may still be present.

Figure S6: Potential outcomes of combining activators and inhibitors. Opacity of image indicates relative concentration of each species.

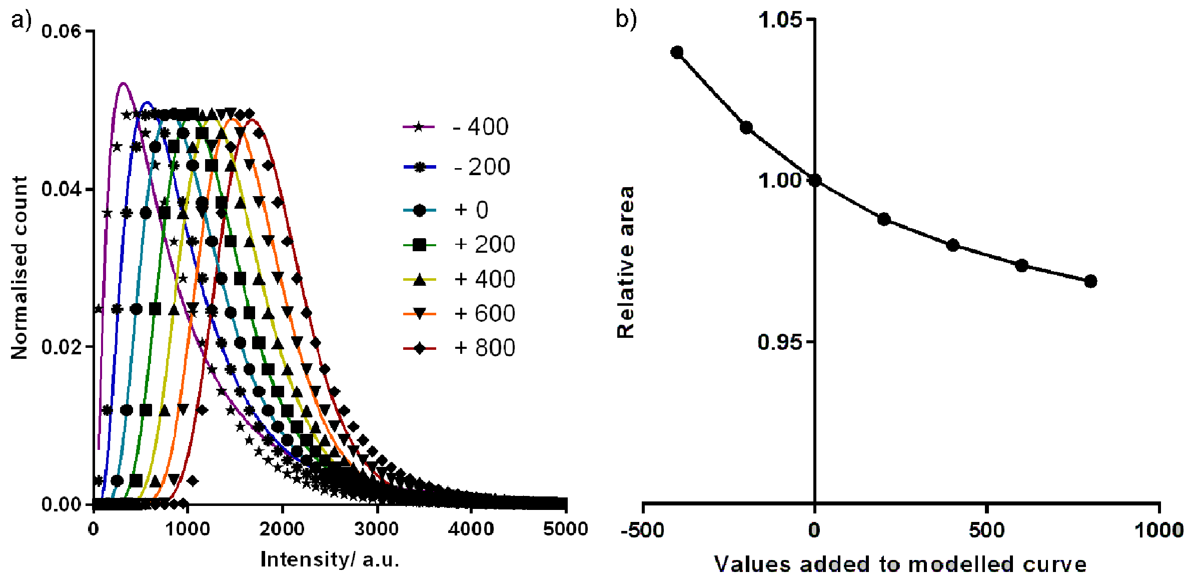

Figure S7: Effect of small x-axis translations on quality of data fit and reported peak areas. a) Data fits of translated idealized data. We modelled an idealized low intensity data peak using parameters based on our experimental results for CD532 (filled circles, aquamarine). Data was translated along the x-axis by amounts indicated in figure legend and fitted to equation (S19). b) Area under each fitted curve in (a), relative to the original curve. Negative x-axis values illustrate effect of curve truncation.
